# Design and Implementation of a Novel Single-Driven Ultrasonic Elliptical Vibration Assisted Cutting Device

**DOI:** 10.3390/mi9100535

**Published:** 2018-10-22

**Authors:** Rongkai Tan, Xuesen Zhao, Xicong Zou, Zengqiang Li, Zhenjiang Hu, Weipan Zhang, Tao Sun

**Affiliations:** 1 Center for Precision Engineering, Harbin Institute of Technology, Harbin 150001, China; tanrongkai17@gmail.com (R.T.); zhaoxuesen@hit.edu.cn (X.Z.); zqlee@hit.edu.cn (Z.L.); huzhenjiang@hit.edu.cn (Z.H.); weipanzhang01@gmail.com (W.Z.); 2 School of Mechatronics Engineering, Heilongjiang University, Harbin 150080, China; zouxicong@hlju.edu.cn

**Keywords:** ultrasonic elliptical vibration cutting device, single driven, micro-dimple patterns, ultrasonic vibration, elliptical vibration cutting

## Abstract

In this paper, a novel single-driven ultrasonic elliptical vibration cutting (SDUEVC) device with a succinct structure and a simple assembly is proposed and investigated. A tailored horn with a tilted-slot structure was employed in the designed SDUEVC device. Also, the elliptical trajectory formation mechanism of the designed SDUEVC device was described by using the theory of mechanical vibration. Furthermore, the finite element method (FEM) was used to optimize the tilted-slot structure parameters and there are four parameters selected as the optimization factors. The results indicated that the proposed SDUEVC device can generate larger vertical amplitude than previous SDUEVC devices, which provides an important and positive effect for the cutting performance of the proposed SDUEVC device. According to the optimized results, a prototype SDUEVC device was fabricated and its vibration characteristic was tested. When the excitation signal voltage was 500 V_p-p_, the test results indicated that the amplitudes in the axial and vertical directions were 8.7 μm and 6.8 μm, respectively. Furthermore, an elliptical trajectory was generated at the cutting tool tip. Finally, the proposed SDUEVC device was used to fabricate microdimple patterns as the initial application to confirm the feasibility of the proposed SDUEVC device.

## 1. Introduction

Ultrasonic elliptical vibration cutting (UEVC) is a promising cutting technique, especially in the precision and ultra-precision machining of difficult-to-cut materials [1], such as hardened steel [2,3,4], tungsten alloy [5], inconel 718 [6,7], tungsten carbide [8,9,10], fiber-reinforced composite [11,12,13], and others [14]. In addition, many researchers have performed in-depth research on UEVC technology for the fabrication of micro-nano structures. Kim et al. [15,16] have investigated the manufacture of micropatterns and micro-V-grooving by using elliptical vibration cutting. Ehmann et al. [17,18,19] proposed a highly efficient machining method (i.e., the elliptical vibrations couple with a high cutting velocity) for rapid generation of microdimples on engineered surfaces. Similarly, Guo et al. [20] have investigated the rapid coloration of metal surfaces by using ultrasonic elliptical vibration texturing. Also, Zhou et al. [21] proposed a double-frequency elliptical vibration cutting method (i.e., elliptical vibration couple with slow slide servo). Kurniawan et al. [22,23] fabricated a two-frequency, elliptical-vibration texturing device and developed a novel surface roughness model for microdimples processed by the double-frequency elliptical vibration cutting technology.

The UEVC device, which is the key component of the UEVC system, has received much attention from researchers. At present, the conventional UEVC devices are mainly achieved by coupling two resonance modes of the device, i.e., longitudinal-longitudinal complex-mode [17], longitudinal-flexural complex-mode [5,24,25,26], and flexural-flexural complex-mode [27,28]. The mode coupling UEVC device usually has two or more groups of piezoelectric (PZT) ceramic pieces, and the PZT ceramic pieces need two ultrasonic power to driven. Furthermore, the phase shift of the excitation signals applied to the PZT ceramic pieces should be controlled for a definite elliptical vibration trajectory. This makes the mode coupling UEVC devices more complicated. Moreover, for the effective excitation of the UEVC device, the resonant frequencies of two resonance modes should be close to each other. However, the resonant mode of the UEVC device is affected by many factors, such as temperature, clamping method, assembling state, material properties, and so on. Hence, it is a great challenge to realize the modal degeneration of the mode-coupling UEVC device.

The single-driven ultrasonic elliptical vibration cutting (SDUEVC) system, which contains a unidirectional vibration transducer, a tailored horn, and one ultrasonic power, is simpler than the mode coupling UEVC system. In addition, the SDUEVC device has a simpler control circuit than the mode coupling UEVC device. Furthermore, the SDUEVC device could be made at lower costs than the mode coupling UEVC device. Therefore, the SDUEVC device is a decent choice for the cases of application that the elliptical vibration trajectory does not require adjustment. Zhang and Li [29] fabricated a SDUEVC device by mounting the mass tool away from the centerline of the vibrator. Their test results indicated that the axial and vertical amplitudes were 16 μm and 2 μm, respectively. Brinksmeier et al. [30] built a similar SDUEVC device, and the maximum amplitude in the vertical direction was 2.5 μm. Zhen et al. [31] explored a SDUEVC device with a complex-beam horn and given the mathematical model of the complex-beam horn. The vibration characteristic test results indicated that the maximum vertical amplitude of the device was 3 μm. 

Therefore, as discussed above, much work has been done by researchers to develop the SDUEVC device. Furthermore, the desirable axial amplitude is easy to achieve for the SDUEVC device. Yet, the previous SDUEVC devices have a serious common disadvantage that the vertical amplitude is small. Ma et al. [22] have investigated the frictional force between the chip and the cutting tool in the UEVC process and pointed out that the time period of friction reversal decreases with the decrement of the vertical amplitude. Moriwaki and Shamoto [27] considered that the friction reversal effect is an important and irreplaceable feature of the UEVC technology. Furthermore, it has been confirmed that the friction reversal effect leads to a significant decrement of chip thickness and average cutting force, a suppression of regenerative chatter, as well as an increase of nominal shear angle [10,27,32,33]. Thus, a novel SDUEVC device with a tilted-slot structure is proposed in this paper, which is able to generate larger vertical amplitude than previous SDUEVC devices by optimizing the tilted-slot structure parameters. Furthermore, the proposed SDUEVC device has a succinct structure and a simple assembly.

The paper is organized as follows. First, the configuration, the elliptical trajectory formation mechanism, and the finite element analysis of the proposed SDUEVC device are studied in Section 2. Section 2.1 describes the configuration of the proposed SDUEVC device. Section 2.2 gives the mathematical model of the elliptical trajectory formation mechanism, while the optimization of the tilted-slot structure parameters by finite element analysis is discussed in Section 2.3. Second, Section 3 describes the vibration characteristic tests of the deigned SDUEVC device and a surface cutting experiment. Finally, conclusions are given in Section 4.

## 2. Design of the Single Driven Ultrasonic Elliptical Vibration Cutting (SDUEVC) Device

### 2.1. Configuration of the SDUEVC Device

**Figure 1:**
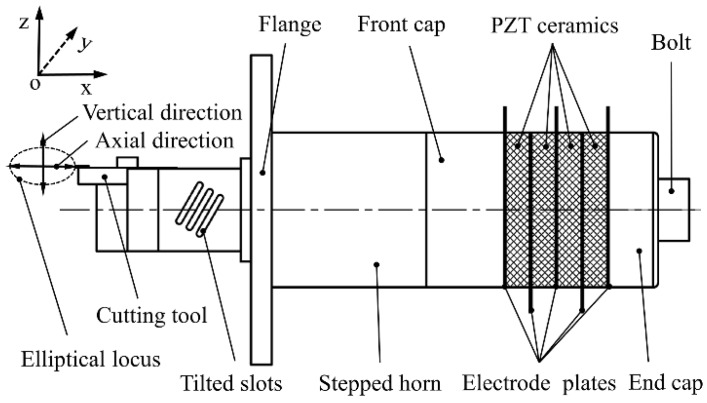
Design of the proposed device.

The configuration of the SDUEVC device is shown in Figure 1, which consists of a longitudinal vibration Langevin transducer and a tailored stepped horn. The longitudinal vibration Langevin transducer, which is assembled first, is the energy conversion part of the SDUEVC device. The transducer included a front cap, an end cap, a binding bolt, electrode plates, and four pieces of PZT ceramics. The PZT ceramics were sandwiched between the front and end caps. It worked in the d33 mode, and was considered to be highly efficient [34]. The tailored stepped horn was used to transfer vibrational energy. In order to achieve a large ratio of amplitude transmission, the horn was stepped at the loop point of the vibration [35]. The flange was located at the vibration node of the vibrator and the cutting tool was mounted at the front end of the stepped horn. As shown in Figure 3, the first step of the stepped horn was a cylindrical structure, while the last step was a rectangular structure for the convenient processing of the tilted slot. When the Langevin transducer was excited by a sinusoidal signal at a specific frequency, the stepped horn with tilted-slot structure made the cutting tool tip to generate an elliptical trajectory in the plane formed by the axial direction and the vertical direction as shown in Figure 1. It is noteworthy that the adjustment of the elliptical locus can been achieved by changing the parameters of the tilted slot and the voltage value of the sinusoidal signal when the excitation frequency was constant.

### 2.2. The Elliptical Trajectory Formation Mechanism 

**Figure 2:**
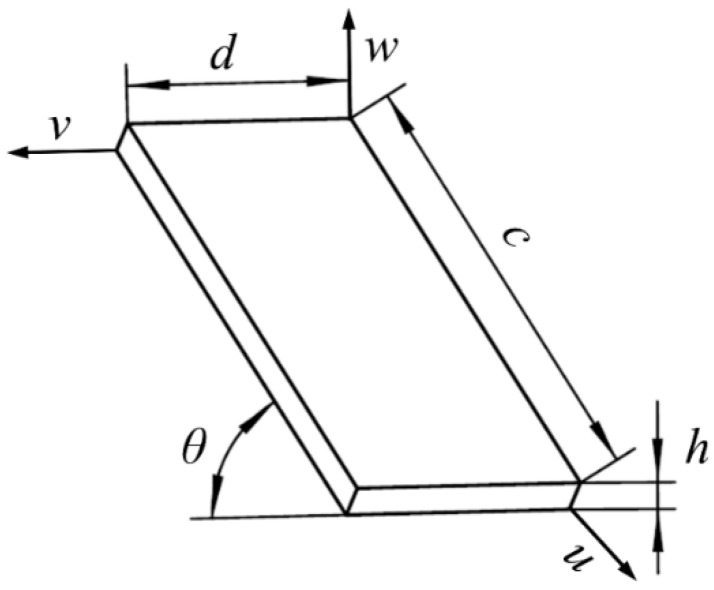
Schematic of the rectangular thin plate.

The tilted-slot structure is the key point to the design of the stepped horn, which makes the longitudinal-bending composite resonant mode of the device inspired. Due to the presence of the tilted slots, a number of inclined rectangular sheets are obtained in the last step of the stepped horn. In order to simplify the dynamic analysis of the thin sheet, it is assumed that the left end of the rectangular thin sheet has only horizontal (i.e., axial direction) displacement, and the right end of the rectangular thin plate is free. Thus, the right end of the thin plate may have a horizontal vibration (*u*) and a vertical vibration displacement (*w*). The schematic of the rectangular thin sheet is shown in Figure 2, where *d* and *c* are the width and length of the rectangular thin sheet, respectively, *h* is the thickness of the rectangular thin sheet, and *θ* is the angle of inclination of the rectangular thin sheet.

For a concise calculation, the rectangular thin plate can be regarded as an elastic thin plate. As a small-deflection elastic thin plate, the Kirchhoff–Love assumptions can be used [36]. The assumptions are as follow: (1) there is no shear stress on the plane parallel to the mid-plane of rectangular thin plate; (2) the thickness of rectangular thin plate is constant; (3) there is no compressive and tensile stress between the planes parallel to the mid-plane of rectangular thin plate, in other words, the normal stress in *w* direction is ignored; and (4) there is no tensile and compression deformation of the mid-plane of rectangular thin plate when the rectangular thin plate undergoes bending deformation. The stepped horn material is titanium alloy (TC4) with a density of *ρ*, an elastic modulus of *E*, and a Poisson’s ratio of *μ*. A Cartesian coordinate system is established on the mid-plane of the rectangular thin plate. Furthermore, *u*, *v*, and *w* are the vibration displacements of the thin plate along the three coordinate axes *x*, *y*, and *z*, respectively. Therefore, the differential equation for the rectangular thin plate under free vibration is as follows:

(1) $$\rho h\frac{\partial^{2}w}{\partial t^{2}}+B\left(\frac{\partial^{4}w}{\partial x^{4}}+2\frac{\partial^{4}w}{\partial x^{2}y^{2}}+\frac{\partial^{4}w}{\partial y^{4}}\right)=0\;$$

(2) $$B=\frac{Eh^{3}}{12(1-\mu^{2})}\;$$

where *B* is the bending stiffness of the rectangular thin plate. According to the assumption that the thin plate is fixed at the left end and the right end is free, the boundary conditions for the free vibration differential equation of the sheet are:

(3) $${\frac{\partial w}{\partial x}|}_{x=0}=0\;$$

(4) $${-B\left(\frac{\partial^{2}w}{\partial x^{2}}+\mu \frac{\partial^{2}w}{\partial y^{2}}\right)|}_{x=c,y=0\;and\;y=d}=0\;$$

Since the deformation of the rectangular thin plate is very small, the corners of the left and right ends of the rectangular thin plate are assumed to be zero. After substitution of Equation (3) and Equation (4) into Equation (1), the solution of Equation (1) is:

(5) $$w=\frac{2c^{2}}{h^{2}}\tan\theta \frac{\partial u\left(x,t\right)}{\partial x}\;$$

Therefore, the horizontal and vertical displacements are:

(6) $$u_{1}=w\sin\theta =\frac{2c^{2}}{h^{2}}\frac{\sin^{2}\theta }{\cos\theta }\frac{\partial u\left(x,t\right)}{\partial x}\;$$

(7) $$w_{1}=w\cos\theta =\frac{2c^{2}}{h^{2}}\sin\theta \frac{\partial u\left(x,t\right)}{\partial x}\;$$

The stepped horn is machined from the same homogeneous material. Then, the equation of wave motion is expressed as:

(8) $$\frac{\partial^{2}u(x,t)}{\partial t^{2}}=\frac{E}{\rho }\frac{\partial^{2}u(x,t)}{\partial x^{2}}\;$$

The general solutions of *u*(*x, t*) have the following form:

(9) $$u\left(x,t\right)=\left[G\cos\left(kx\right)+H\sin\left(kx\right)\right]e^{j\omega t}\;$$

where G and H are constants, *k* is circular wavenumber, *k* = ω/*v*_0_, ω = 2π*f*, *v*_0_ is the propagation speed of the sound wave in titanium alloy (TC4), and *f* is the resonance frequency of the stepped horn. When both ends of the stepped horn are free, the value of H is zero. Furthermore, when the length of the first and last steps of the stepped horn are equal (*l* is half the length of the stepped horn), Equation (9) can be written as:

(10) $$u\left(x,t\right)=G\cos\left(\frac{n\pi }{2l}x\right)e^{j\omega t}\;$$

Thus, the horizontal and vertical displacements can be expressed as:

(11) $$u_{1}\left(x,t\right)=\frac{2c^{2}Gk}{h^{2}}\frac{\sin^{2}\theta }{\cos\theta }\sin\left(kx\right)e^{j\omega t}\;$$

(12) $$w_{1}\left(x,t\right)=\frac{2c^{2}Gk}{h^{2}}\sin\theta \sin\left(kx\right)e^{j\omega t}\;$$

It is obvious that there are both bending vibration and axial vibration at the right end of the rectangular thin plate. Therefore, when a single longitudinal harmonic excitation signal was applied to the stepped horn with the tilted slot, the stepped horn was excited by both vertical and horizontal vibrations due to the effect of the tilted slot. Due to the influence of the damping of two vibration directions and natural vibration characteristics, the vibration response phase of vertical and horizontal directions was different, and the trajectory of the composite vibration was elliptical. 

### 2.3. Finite Element Analysis of the SDUEVC Device

**Figure 3:**
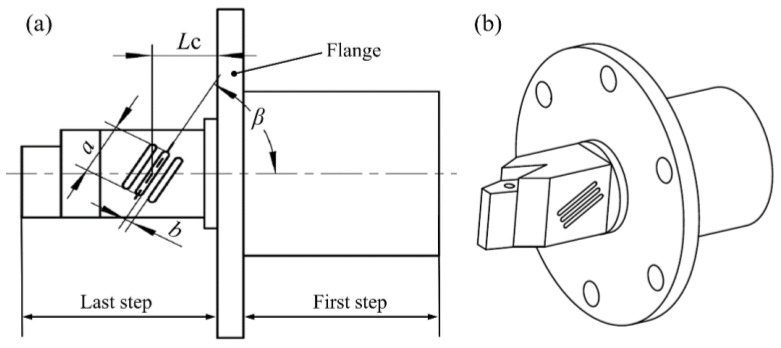
(**a**) Front view of the stepped horn. (**b**) Axonometric drawing of the stepped horn.

The parameters of the tilted slot and the voltage value of the excitation signal are two main factors that determine the elliptical trajectory of the cutting tool tip. The voltage value of the excitation signal can be adjusted during the cutting experiment, while the parameters of the tilted slot is not changeable when the stepped horn was processed. Therefore, the optimization of the tilted-slot structure parameters is indispensable for a desirable value of the vertical amplitude. As discussed in Section 1, the vertical amplitude is a significant parameter for the cutting performance of the designed SDUEVC. The schematic of the stepped horn is shown in Figure 3. The tilted slots have been machined in the last step of the stepped horn. There are four parameters of the tilted slot structure selected as the optimization factors, where *β* is the slope angle of the tilted slot, *L*_C_ is the distance from the tilted slot to flange, *b* is the spacing of the tilted slots, and *a* is the length of the tilted slot. 

The finite element method (FEM) was used to perform modal analysis and transient kinetic analysis of the proposed SDUEVC device. A combined parameter of the tilted slot was firstly selected. Then, the resonant frequency could be determined by performing the modal analysis of the device with the combined parameters. Finally, a sinusoidal displacement signal with the resonant frequency of the device was applied to the large end face of the stepped horn. Furthermore, the amplitude of the sinusoidal displacement signal was set as 5 μm. The axial and vertical vibration amplitudes of the cutting tool tip were extracted and recorded. A SOLID227 element (ANSYS 14.0) was used for meshing. The electrode sheets were overlooked because of the very small mass. Titanium alloy (TC4) was the selected material of the stepped horn (mass density *ρ* = 4450 kg/m^3^, Poisson ratio *σ* = 0.34, Young’s modulus *E* = 11 × 10^10^ N/m^2^). The material of the front and end caps was selected as aluminum alloy 6061 (mass density *ρ* = 2700 kg/m^3^, Poisson ratio *σ* = 0.33, Young’s modulus *E* = 6.89 × 10^10^ N/m^2^). The material of the bolt was selected as steel 45 (*ρ* = 7800 kg/m^3^, *E* = 2.1 × 10^11^ N/m^2^, *σ* = 0.31). The PZT ceramics material was PZT-81(*ρ* = 7500 kg/m^3^, *E* = 8.3 × 10^10^ N/m^2^, *σ* = 0.3), and the physical parameters of the PZT-81 material were as follows:

(13) $$d=\left[\begin{array}{cc} \begin{array}{ccc} 0 & 0 & 0\\ 0 & 0 & 0\\ -0.93 & -0.93 & 2.18 \end{array} & \begin{array}{ccc} 0 & 3.3 & 0\\ 3.3 & 0 & 0\\ 0 & 0 & 0 \end{array} \end{array}\right]\times 10^{-10}\;C/N,$$

(14) $$C^{E}=\left[\begin{array}{cccccc} 14.9 & 8.11 & 8.11 & 0 & 0 & 0\\ 8.11 & 14.9 & 8.11 & 0 & 0 & 0\\ 8.11 & 0.11 & 13.2 & 0 & 0 & 0\\ 0 & 0 & 0 & 3.13 & 0 & 0\\ 0 & 0 & 0 & 0 & 3.13 & 0\\ 0 & 0 & 0 & 0 & 0 & 3.4 \end{array}\right]\times 10^{10}\;{N/m}^{2},$$

(15) $$\varepsilon^{T}=\left[\begin{array}{ccc} 8.0 & 0 & 0\\ 0 & 8.0 & 0\\ 0 & 0 & 5.3 \end{array}\right]\times 10^{-9}\;F/m.$$

**Figure 4:**
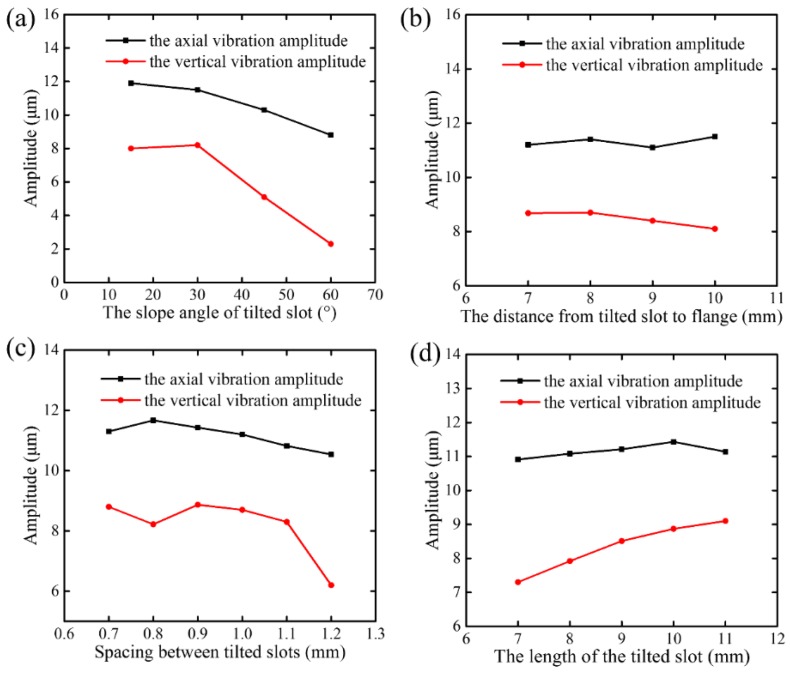
The effects of tilted slot parameters on vibration amplitudes of axial and vertical directions. (**a**) Effects of the slope of tilted slot on vibration amplitudes (*a* = 10 mm, *b* = 1.0 mm, *L*_C_ = 10 mm). (**b**) Effects of the distance from tilted slot to flange on vibration amplitudes (*a* = 10 mm, *b* = 1.0 mm, *β* = 30°). (**c**) Effects of the spacing of the tilted slots on vibration amplitudes (*a* = 10 mm, *L*_C_ = 8 mm, *β* = 30°). (**d**) Effects of the length of tilted slot on vibration amplitudes (*b* = 0.9 mm, *L*_C_ = 8 mm, *β* = 30°).

**Figure 5:**
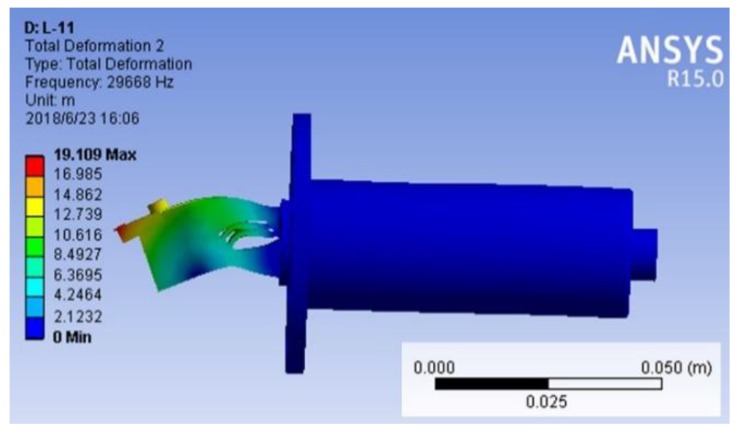
Vibration mode of the designed single driven ultrasonic elliptical vibration cutting (SDUEVC) device.

Figure 4 records the vibration amplitudes of axial and vertical directions at varied structure parameters of the tilted slot. Figure 4a shows that as the slope angle of the tilted slot (*β*) increased, the axial amplitude decreased, while the amplitude in the vertical direction first increased and then decreased. Furthermore, when the slope angle of the tilted slot was greater than 30°, the amplitude in the vertical direction decreased sharply. Therefore, the slope angle of the tilted slot was set as 30°. The effects of the distance from the tilted slot to the flange (*L*_C_) on the vibration amplitudes of the axial and vertical directions are shown in Figure 4b. The axial amplitude remained about constant, and the vertical amplitude started to decrease when the distance exceeded 8 mm. Therefore, the distance from the tilted slot to the flange was selected as 8 mm. Figure 4c shows that as the spacing of the tilted slots (*b*) exceeded 0.9 mm, the reduction rates of the axial amplitude and vertical amplitude were almost at the same level, and the vertical amplitude began to decrease rapidly when *b* was greater than 1.1 mm. It was obvious that the vertical amplitude had the largest value when the spacing of tilted slots was 0.9 mm. As shown in Figure 4d, the axial amplitude remained about constant, and the vertical amplitude gradually increased with an increase of the length of tilted slot. Therefore, the length of the tilted slot was set as 11 mm. Furthermore, it could not be further increased due to the limitation of the overall structure. It is worth noting that the sensitivity of vertical amplitude to the parameters of the tilted-slot structure was greater than the axial amplitude. Besides, the slope angle of tilted slot and the spacing of tilted slots had a conspicuous influence on the vertical amplitude, especially when the slope angle of tilted slot was greater than 30° or the spacing of tilted slots was greater than 1.1 mm. The simulation results showed that the optimized amplitudes in the axial and vertical directions were 11.1 μm and 9.1 μm, respectively. That is to say, a desirable vertical amplitude was obtained by optimizing the parameters of the tilted slot. Based on the optimized results of the tilted-slot structure, a three-dimensional model of the proposed SDUEVC device with new structural parameters was applied to modal analysis. The results of modal analysis showed that the longitudinal and bending compound vibration mode was inspired and the resonant frequency was 29,668 Hz, as shown in Figure 5.

## 3. Results and Discussion

### 3.1. Experiment to Test the Vibration Characteristics of the SDUEVC Device

**Figure 6:**
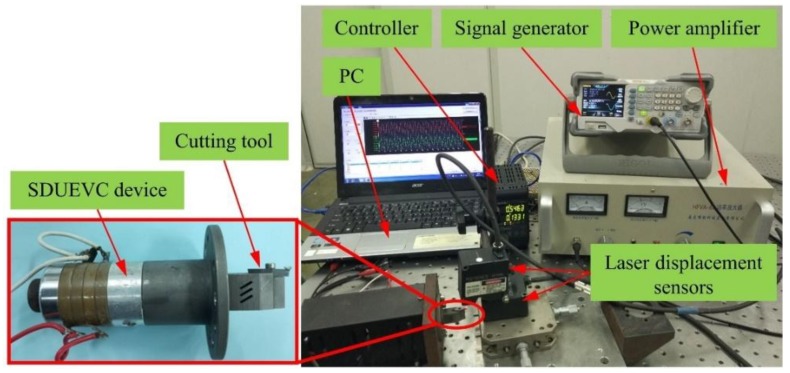
Designed SDUEVC device and the vibration characteristics testing instruments.

In order to validate the mathematical model of the elliptical trajectory formation mechanism of the designed SDUEVC device and verify the results of numerical modelling, a SDUEVC device with optimized dimensions was fabricated. The vibration characteristics of the SDUEVC device were tested, and the turning performance in the practical application was demonstrated. The designed SDUEVC device and the vibration characteristics testing instruments are shown in Figure 6. The two laser displacement sensors were perpendicular to the cutting tool tip in the axial and vertical directions. The vibration data were recorded by the controller and displayed on the personal computer. The sampling frequency of the controller was selected as 392 kHz and the measurement accuracy of laser displacement sensors was 0.1 μm to meet the testing requirement. The excitation signal applied to the SDUEVC device was generated by a signal generator and a power amplifier. The frequency of the excitation signal can be adjusted by the signal generator while the voltage value could be adjusted by the power amplifier.

**Figure 7:**
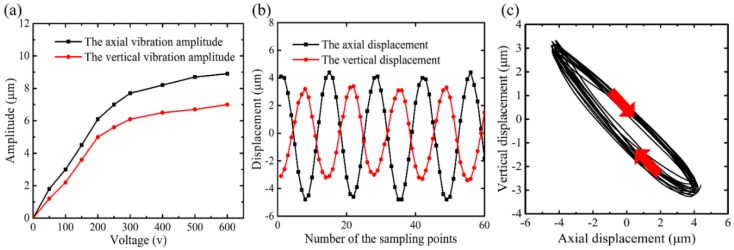
The vibration performance testing results of SDUEVC device. (**a**) The amplitudes in axial and vertical directions of SDUEVC device with different excitation signal voltages. (**b**) The recorded vibration displacements of the tool tip (the excitation signal voltages was 500 V_p-p_). (**c**) The vibration trajectory of the tool tip.

Prior to the vibration characteristics test, the resonance frequency of the fabricated SDUEVC device was tested. The result showed the resonance frequency of the SDUEVC device was 28.3 kHz. The discrepancy between the tested result and the simulation may primarily be ascribed to the neglect of electrode sheets as well as the differences between the real materials and the ideal models. In the following experiments, the frequency of excitation signal was set as 28.3 kHz. Figure 7 shows the vibration characteristics of the designed SDUEVC device. The amplitudes in the axial and vertical directions of the SDUEVC device with different voltages of the excitation signal are plotted in Figure 7a. When the voltage of the excitation signal was less than 250 V_p-p_, both the axial and vertical vibration amplitudes increased approximately linearly with the voltage value of the excitation signal, and the axial amplitude was always greater than the vertical amplitude. However, both the axial and vertical amplitude curves became flat when the voltage of the excitation signal was greater than 300 V_p-p_. Furthermore, the vibration amplitudes of the tool tip and the voltage value of the excitation signal were positively related. Figure 7b records the axial and vertical vibration displacements of the tool tip when the excitation signal voltages was 500 V_p-p_. The amplitudes in the axial and vertical directions were 8.7 μm and 6.8 μm, respectively. The trajectory of the tool tip could be fitted into an ellipse, as shown in Figure 7c. The results of the vibration characteristics experiment indicated that the maximum vertical amplitude of the designed SDUEVC device was 7.1 μm when the excitation signal voltage was 600 V_p-p_. Furthermore, an elliptical trajectory was generated at the cutting tool tip.

### 3.2. Cutting Experiments of the Proposed SDUVEC Device

**Figure 8:**
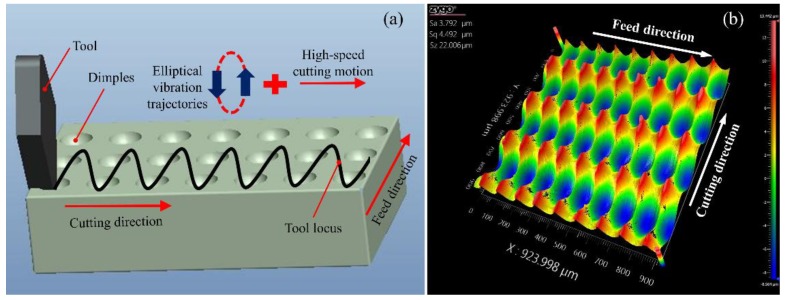
Application of the elliptical vibration cutting for surface topography cutting. (**a**) Schematic of elliptical vibration cutting for rapid fabrication of microdimple array. (**b**) The machined surface measured using a Zygo white-light interferometer.

To confirm the feasibility of the proposed SDUVEC device in the actual application, the SDUVEC device was applied to fabricate microdimple patterns as the initial application. When the elliptical vibration trajectory was coupled with a high cutting velocity, the tool trajectory could create the controllable dimples on the workpiece. The principle of the cutting process is shown in Figure 8a. In the cutting experiment, the machine tool only provides two linear motions, namely feed motion and cutting motion, the SDUEVC device provided a microscale elliptical motion at the tip of the tool. When the elliptical vibration trajectory and the depth of cut were constant, the dimple spacing in the cutting direction could be adjusted using the nominal cutting velocity and the dimple spacing in the feed direction can be adjusted using the feed velocity. Furthermore, the shape of the dimple was determined using the cutting parameters, vibration parameters (i.e., elliptical trajectory and vibration frequency), and tool parameters. Compared to the existing processing technologies for the fabrication of microdimples, the ultrasonic elliptical vibration turning process was more flexibility and had a higher efficiency. The workpiece material was aluminum alloy. The experimental parameters are listed in Table 1. The machined surface was characterized using a Zygo white-light interferometer (Zygo Newview 8200 from Zygo Crop., Berwyn, PA, USA). As shown in Figure 8b, the regular microdimple array was obtained on the workpiece surface, which indicated that the designed SDUEVC device worked stably in the actual cutting experiment.

## 4. Conclusions

Based on the design analysis, the simulation optimization and the performance verification experiments of the proposed SDUEVC device, the following conclusions can be drawn: 

(1) An innovative SDUEVC device was proposed and investigated in this paper. The proposed SDUEVC device had a succinct structure and a simple assembly. It consisted of a longitudinal vibration Langevin transducer and a tailored horn with a tilted-slot structure. The tilted-slot structure was the key point to the design of the horn, which made the longitudinal-bending composite resonant mode of the device inspired. A mathematical model of the elliptical trajectory formation mechanism of the designed SDUEVC device was established, which provided a theoretical basis for the realization of the SDUEVC device.

(2) The FEM simulation results showed that the sensitivity of vertical amplitude to the parameters of the tilted slot structure was greater than the axial amplitude. Furthermore, the slope angle of the tilted slot and the spacing of the tilted slots had a great influence on the vertical amplitude, especially when the slope angle of the tilted slot was greater than 30° or the spacing of the tilted slots was greater than 1.1 mm. Also, the optimized amplitudes in the axial and vertical directions were 11.1 μm and 9.1 μm, respectively. Furthermore, the longitudinal and bending compound resonant mode of the proposed SDUEVC device was inspired. 

(3) The vibration performance test results showed that the vibration amplitudes of the tool tip had a strong correlation with the voltage value of the excitation signal and an elliptical trajectory was obtained at the cutting tool tip. In addition, when the excitation signal voltage was 500 V_p-p_, the amplitudes in the axial and vertical directions were 8.7 μm and 6.8 μm, respectively. Furthermore, when the excitation signal voltage was 600 V_p-p_, the maximum vertical amplitude of the designed SDUEVC device was 7.1 μm, which meant the proposed SDUEVC device had generated a larger vertical amplitude than previous SDUEVC devices.

To demonstrate the feasibility of the proposed SDUEVC device, the device was applied to fabricate microdimple patterns. The regular microdimples were obtained on a workpiece surface, which indicated the designed SDUEVC device worked stably in the actual cutting process.

## Figures and Tables

**Table 1:** Turning parameters.

Vibration conditions	Frequency (kHz)	28.3
	Axial amplitude (μm)	8.7
	Vertical amplitude (μm)	6.8
Cutting conditions	Nominal cutting speed (m/s)	5.8
	Feed speed (μm/s)	100
	Nominal cutting depth (μm)	2
Cutting tool	Material	SCD
	Normal rake angle (°)	0
	Normal clearance angle (°)	11
	Nose radius (mm)	0.2

## References

[1] Zhang J., Cui T., Ge C., Sui Y., Yang H. (2016). Review of micro/nano machining by utilizing elliptical vibration cutting. Int. J. Mach. Tools Manuf..

[2] Shamoto E., Moriwaki T. (1999). Ultaprecision diamond cutting of hardened steel by applying elliptical vibration cutting. CIRP Ann.-Manuf. Technol..

[3] Brinksmeier E., Gläbe R. (2001). Advances in precision machining of steel. CIRP Ann.-Manuf. Technol..

[4] Zhang X., Kumar A.S., Rahman M., Nath C., Liu K. (2010). Experimental study on ultrasonic elliptical vibration cutting of hardened steel using PCD tools. J. Mater. Process. Technol..

[5] Suzuki N., Haritani M., Yang J., Hino R., Shamoto E. (2007). Elliptical vibration cutting of tungsten alloy molds for optical glass parts. CIRP Ann.-Manuf. Technol..

[6] Zhao H., Li S., Zou P., Kang D. (2017). Process modeling study of the ultrasonic elliptical vibration cutting of Inconel 718. Int. J. Adv. Manuf. Technol..

[7] Lotfi M., Amini S. (2018). FE simulation of linear and elliptical ultrasonic vibrations in turning of Inconel 718. Proc. Inst. Mech. Eng. Part E-J. Process Mech. Eng..

[8] Zhang J., Suzuki N., Wang Y., Shamoto E. (2014). Fundamental investigation of ultra-precision ductile machining of tungsten carbide by applying elliptical vibration cutting with single crystal diamond. J. Mater. Process. Technol..

[9] Nath C., Rahman M., Neo K.S. (2009). A study on ultrasonic elliptical vibration cutting of tungsten carbide. J. Mater. Process. Technol..

[10] Nath C., Rahman M., Neo K.S. (2009). Machinability study of tungsten carbide using PCD tools under ultrasonic elliptical vibration cutting. Int. J. Mach. Tools Manuf..

[11] Xu W., Zhang L.C., Wu Y. (2014). Elliptic vibration-assisted cutting of fibre-reinforced polymer composites: Understanding the material removal mechanisms. Compos. Sci. Technol..

[12] Xu W.X., Zhang L.C. (2015). Ultrasonic vibration-assisted machining: Principle, design and application. Adv. Manuf..

[13] Xu W.X., Zhang L.C. (2016). Mechanics of fibre deformation and fracture in vibration-assisted cutting of unidirectional fibre-reinforced polymer composites. Int. J. Mach. Tools Manuf..

[14] Song Y.C., Park C.H., Moriwaki T. (2010). Mirror finishing of Co–Cr–Mo alloy using elliptical vibration cutting. Precis. Eng..

[15] Kim G.D., Loh B.G. (2011). Direct machining of micro patterns on nickel alloy and mold steel by vibration assisted cutting. Int. J. Precis. Eng Manuf..

[16] Kim G.D., Loh B.G. (2008). Characteristics of elliptical vibration cutting in micro V-grooving with variations of elliptical cutting locus and excitation frequency. J. Micromech. Microeng..

[17] Guo P., Ehmann K.F. (2013). Development of a tertiary motion generator for elliptical vibration texturing. Precis. Eng..

[18] Guo P., Ehmann K.F. (2013). An analysis of the surface generation mechanics of the elliptical vibration texturing process. Int. J. Mach. Tools Manuf..

[19] Zhang C., Shi G., Ehmann K.F., Li Y. (2016). Modeling and simulation of micro-groove topography on cylindrical surface by elliptical vibration-assisted turning. Int. J. Adv. Manuf. Technol..

[20] Yang Y., Pan Y., Guo P. (2017). Structural coloration of metallic surfaces with micro/nano-structures induced by elliptical vibration texturing. Appl. Surf. Sci..

[21] Zhou X., Zuo C., Liu Q., Lin J. (2016). Surface generation of freeform surfaces in diamond turning by applying double-frequency elliptical vibration cutting. Int. J. Mach. Tools Manuf..

[22] Kurniawan R., Ko T.J., Li C.P., Kumaran S.T., Kiswanto G., Guo P., Ehmann K.F. (2017). Development of a two-frequency, elliptical-vibration texturing device for surface texturing. J. Mech. Sci Technol..

[23] Kurniawan R., Kiswanto G., Ko T.J. (2017). Surface roughness of two-frequency elliptical vibration texturing (TFEVT) method for micro-dimple pattern process. Int. J. Mach. Tools Manuf..

[24] Zhou M., Hu L. (2015). Development of an innovative device for ultrasonic elliptical vibration cutting. Ultrasonics.

[25] Amini S., Khosrojerdi M.R., Nosouhi R. (2015). Elliptical ultrasonic–assisted turning tool with longitudinal and bending vibration modes. Proc. Inst. Mech. Eng. Part B-J. Eng. Manuf..

[26] Tan R., Zhao X., Zou X., Sun T. (2018). A novel ultrasonic elliptical vibration cutting device based on a sandwiched and symmetrical structure. Int. J. Adv. Manuf. Technol..

[27] Moriwaki T., Shamoto E. (1995). Ultrasonic elliptical vibration cutting. CIRP Ann.-Manuf. Technol..

[28] Huang W.H., Yu D.P., Zhang M., Ye F.G., Yao J. (2017). Analytical design method of a device for ultrasonic elliptical vibration cutting. J. Acoust. Soc. Am..

[29] Li X., Zhang D. (2006). Ultrasonic elliptical vibration transducer driven by single actuator and its application in precision cutting. J. Mater. Process. Technol..

[30] Brinksmeier E., Gläbe R. (1999). Elliptical vibration cutting of steel with diamond tools. Proc. ASPE.

[31] Yin Z., Fu Y., Xu J., Li H., Cao Z., Chen Y. (2017). A novel single driven ultrasonic elliptical vibration cutting device. Int. J. Adv. Manuf. Technol..

[32] Ma C., Ma J., Shamoto E., Moriwaki T. (2011). Analysis of regenerative chatter suppression with adding the ultrasonic elliptical vibration on the cutting tool. Precis. Eng..

[33] Shamoto E., Suzuki N., Hino R. (2008). Analysis of 3D elliptical vibration cutting with thin shear plane model. CIRP Ann.-Manuf. Technol..

[34] Yang X., Liu Y., Chen W., Liu J. (2013). Longitudinal and bending hybrid linear ultrasonic motor using bending PZT elements. Ceram. Int..

[35] Watanabe Y., Tsuda Y., Mori E. (1991). A study on a transducer-stepped type solid horn system for flexural mode ultrasonic high power transducer with one dimensional construction. Ultrason. Int..

[36] Parkus H. (1976). Thermoelasticity.

